# High Na^+^ Environments Impair Phagocyte Oxidase-Dependent Antibacterial Activity of Neutrophils

**DOI:** 10.3389/fimmu.2021.712948

**Published:** 2021-09-10

**Authors:** Luka Krampert, Katharina Bauer, Stefan Ebner, Patrick Neubert, Thomas Ossner, Anna Weigert, Valentin Schatz, Martina Toelge, Agnes Schröder, Martin Herrmann, Markus Schnare, Anca Dorhoi, Jonathan Jantsch

**Affiliations:** ^1^Institute of Clinical Microbiology and Hygiene, University Hospital of Regensburg and University of Regensburg, Regensburg, Germany; ^2^Max Planck Institute (MPI) of Biochemistry, Martinsried, Germany; ^3^Institute of Orthodontics, University Hospital of Regensburg, Regensburg, Germany; ^4^Department of Internal Medicine 3-Rheumatology and Immunology and Deutsches Zentrum für Immuntherapie, Friedrich-Alexander-University Erlangen-Nürnberg, University Hospital Erlangen, Erlangen, Germany; ^5^Department of Immunology, Philipps University Marburg, Marburg, Germany; ^6^Institute of Immunology, Friedrich-Loeffler Institut, Greifswald, Germany; ^7^Faculty of Mathematics and Natural Sciences, University of Greifswald, Greifswald, Germany

**Keywords:** sodium, phagocyte oxidase, infection, neutrophils, reactive oxygen species

## Abstract

Infection and inflammation can augment local Na^+^ abundance. These increases in local Na^+^ levels boost proinflammatory and antimicrobial macrophage activity and can favor polarization of T cells towards a proinflammatory Th17 phenotype. Although neutrophils play an important role in fighting intruding invaders, the impact of increased Na^+^ on the antimicrobial activity of neutrophils remains elusive. Here we show that, in neutrophils, increases in Na^+^ (high salt, HS) impair the ability of human and murine neutrophils to eliminate *Escherichia coli* and *Staphylococcus aureus*. High salt caused reduced spontaneous movement, degranulation and impaired production of reactive oxygen species (ROS) while leaving neutrophil viability unchanged. High salt enhanced the activity of the p38 mitogen-activated protein kinase (p38/MAPK) and increased the interleukin (IL)-8 release in a p38/MAPK-dependent manner. Whereas inhibition of p38/MAPK did not result in improved neutrophil defense, pharmacological blockade of the phagocyte oxidase (PHOX) or its genetic ablation mimicked the impaired antimicrobial activity detected under high salt conditions. Stimulation of neutrophils with phorbol-12-myristate-13-acetate (PMA) overcame high salt-induced impairment in ROS production and restored antimicrobial activity of neutrophils. Hence, we conclude that high salt-impaired PHOX activity results in diminished antimicrobial activity. Our findings suggest that increases in local Na^+^ represent an ionic checkpoint that prevents excessive ROS production of neutrophils, which decreases their antimicrobial potential and could potentially curtail ROS-mediated tissue damage.

## Introduction

High salt diets (1, 2), renal impairment (3–6), inflammation, and infection (7–11) can induce Na^+^ accumulation in skin tissues that can be simulated by addition of approximately 40 mM NaCl to standard cell culture media (= high salt condition, HS) (2, 8, 12, 13). In addition to skin, other organs such as liver, spleen and thymus can display enhanced Na^+^ levels [reviewed in: (14)]. Although the mechanisms that govern local Na^+^ accumulation are still elusive [reviewed in: (14)], it became clear in the last years that these increases in Na^+^ and other ions impact the biology of various immune cells [reviewed in: (14–19)] and serve as ‘ionic checkpoints’ (20) in immunity.

Increases in Na^+^ favor the polarization of T cells towards an inflammatory Th17 phenotype (10, 21–23). In macrophages, HS in combination with inflammatory stimuli leads to enhanced pro-inflammatory activation and increased antimicrobial capacity (8, 12, 13, 24, 25), while limiting their regulatory features (26–29). Mechanistically, this HS-boosted proinflammatory macrophage activation requires osmoprotective signaling involving the mitogen-activated protein kinase (MAPK) p38 and the transcription factor ‘nuclear factor of activated T cells 5’ (NFAT5/TonEBP), an emerging modulator in immunity [reviewed in: (30, 31)]. This signaling circuit is crucial in renal defenses against uropathogenic *Escherichia coli* [UPEC, (32)] under regular diet conditions as well as in cutaneous macrophage-driven antimicrobial responses against the protozoan parasite *Leishmania major* under experimental HS diet (8).

Effects of HS on polymorphonuclear leukocytes (PMN) are less clear. PMN are promptly recruited to the site of infection and contribute to warding off pathogens [reviewed in: (33)] but also to pathology [reviewed in: (34)]. Several studies reveal that HS can boost PMN activity (35–37), whereas other results indicate impairment in inflammatory activity and activation of PMN (35, 38–45). In line with the disparate findings regarding the impact of HS on neutrophil activation, the effects of HS on antibacterial capacity of PMN are not fully understood. Increases in extracellular Na^+^ either impair (46, 47), augment (36) or do not affect the antimicrobial activity of neutrophils (25). Moreover, the underlying mechanisms contributing to these divergent outcomes remain elusive.

Here, we show that HS diminishes the antimicrobial activity of human and murine PMN against *E. coli*. Mechanistically, reduced bactericidal activity is due to impairment of the neutrophil enzyme nicotinamide adenine dinucleotide phosphate (NADPH) oxidase (PHOX) and subsequently reduced ROS production.

## Material and Methods

### Reagents and Antibodies

NaCl was purchased from Merck. *E. coli* (HB101 laboratory strain) (48) and *E. coli-GFP* harboring pWRG167 (12) were kept on Mueller Hinton agar II plates. *S. aureus* (ATCC 29213) was kept on Columbia sheep blood plus agar plates. Bacterial overnight liquid cultures were generated in LB media. All *in vitro* experiments were performed in RPMI media (Gibco; # 618700044) containing 10% fetal calf serum (FCS; Sigma, #F7524). We purchased phorbol-12-myristate-13-acetate (PMA; #P1585), N-formylmethionyl-leucyl-phenylalanine (fMLP; #F3506), and diphenyleneiodonium chloride (DPI; #D2926) from Sigma and SB203580 (#tlrl-sb20) from Invivogen. For western blotting, we used rabbit-anti-phospho-p38 mouse (Cell Signaling Technology, #4511S) and mouse-anti-Vinculin (BioRad, #V284) primary antibodies. As secondary antibody, we used HRP-conjugated swine-anti-rabbit and goat-anti-mouse antibodies (Dako). For flow cytometry, we stained cells using Brilliant violet 421 anti-human CD62L (BioLegend, #304828), APC anti-human CD11b (BioLegend, #301350), Pacific-Blue anti-human CD66b (BioLegend, #305112), PE anti-human CD35 (BioLegend, #333406) antibodies. For confocal microscopy, we used the rabbit-anti-myeloperoxidase (Agilent, #A039829-2) primary antibody, the donkey-anti-rabbit-Cy5 conjugated (Dianova, #711-606-152) secondary antibody and the Phalloidin AF-647 (Thermo Fisher, #A22287) marker.

### PMN Isolation and Infection

We obtained peripheral blood from healthy donors and employed established standard procedures (49–51) in order to isolate PMN from peripheral blood by density gradient centrifugation using Lymphoflot (Bio-Rad, #824012). After hypotonic lysis of erythrocytes, the remaining PMN were adjusted to 250 000 cells per well and incubated for 1 h at 37°C, 5% CO_2_ in 24-well plates. If DPI was used, 3 µM of DPI was added 30 min prior to stimulation. Subsequently, 40 mM NaCl (= high salt, HS) or mannitol was added where indicated. Together with the standard cell culture media conditions (= normal salt, NS), they were additionally incubated for 1 h. Cells were infected with *E. coli* at a multiplicity of 1 (MOI 1) and centrifuged to synchronize the infection. For infection with *S. aureus* (MOI 1), bacteria were opsonized with 20% human serum AB male (Biowest). Where indicated, 10 ng/ml PMA was added at this stage. Infection was terminated after 1 h - 1.5 h by addition of 400 µl cold PBS. Subsequently, serial dilutions were generated and plated on Mueller Hinton agar II plates. After overnight incubation, colony forming units (CFU) were counted and normalized to the mean of the respective NS group.

### PBMC Isolation and Infection

We obtained peripheral blood from healthy donors and isolated peripheral blood mononuclear cells (PBMC) by density gradient centrifugation using Lymphoflot (Bio-Rad). PBMC were washed twice with PBS and adjusted to 250 000 cells per well in 24-well plates. PBMC infection was performed as described previously in murine macrophages (12). Briefly, cells were infected with *E. coli* (MOI 100) and subjected to gentamicin protection assays. After 2 h of infection, cells were lysed with PBS containing 0.1% Triton-X (PanReac AppliChem, #9036-19-5) and 0.05% Tween-80 (Sigma, #8.22187.0500) and serial dilutions were plated on Mueller Hinton agar II plates. CFU were counted and normalized to the mean of the respective NS group.

### Murine Granulocyte Isolation and Infection

We obtained femurs and tibiae from C57BL/6 wildtype (WT) and *Cybb* ^-/-^ mice (including the respective controls) (52) and flushed the bone marrow using microlances (13 gauge, BD) and syringes (BD). Erythrocyte lysis was performed using ACK buffer (0.1 mM EDTA, 10 mM KHCO_3_, 150 mM NH_4_Cl, pH 7.3-7.5 in H_2_O_dd_) for 10 min at room temperature. Subsequently, cells were washed and passed through a 100 µm cell strainer, before they were resuspended in MACS buffer (0.5% BSA, 2 mM EDTA, pH 7.2 in PBS). We purified neutrophils by negative magnetic selection using the ‘Neutrophil isolation Kit’ (Miltenyi Biotec, #130-097-658) according to the manufacturer’s instructions. Infection of murine granulocytes was performed as described above but with MOI 0.1.

### BMDM Generation and Infection

Bone marrow-derived macrophages (BMDM) were generated in Teflon-bags using L929 supernatant as described earlier (53). Infection experiments (MOI 100) were performed as described above or previously (12).

### Peritoneal Cell Purification and Infection

We induced a sterile inflammatory response by injecting thioglycollate (Sigma, #B2551) in the peritoneal cavity (54, 55). PMN were isolated with cold PBS from the peritoneum one day after injection. Erythrocyte lysis was performed using ACK buffer for 10 min at room temperature. Subsequently, cells were washed and PMN infection experiments were performed as described earlier. 4 days after induction of peritoneal inflammation by injection of thioglycollate, peritoneal macrophages (pMΦ) were obtained essentially as described earlier (56, 57). After 10 min of erythrocyte lysis using ACK buffer at room temperature, peritoneal cells were washed, seeded and incubated for 1-2 h before we eliminated nonadherent cells by washing. Then macrophage infection experiments were performed as described previously (12).

### Immunofluorescence Microscopy

PMN were isolated as described above and seeded on gelatin/fibronectin (Sigma, #F1141) pre-coated coverslips. After 2 h of incubation, cells were infected as described earlier, but with *E. coli-GFP* (MOI 1). 1 h post infection, cells were fixed with 4% PFA (Sigma, #1.04003.1000; diluted in PBS) overnight. The next day, cells were permeabilized (PBS with 2% BSA and 0.1% Saponin) and stained with anti-Phalloidin AF-647 antibody for 1 h, followed by 30 min incubation with the secondary antibody and subsequent mounting of the slides with Prolong Gold containing DAPI (Invitrogen, #P36931). Images were collected with a Leica TCS SP5 confocal laser scanning microscope and processed using the Leica Application Suite (version 2.7.3.9723). Bacterial load was analyzed in Fiji (58).

### Analysis of Neutrophil Extracellular Traps

Formation of neutrophil extracellular traps (NETs) was analyzed *via* confocal microscopy, as described elsewhere (59) with minor modifications. PMN were isolated and seeded on gelatin/fibronectin pre-coated coverslips. Subsequently, 40 mM NaCl was added to the HS conditions and, together with the NS conditions, cells were additionally incubated for 1 h. Then, cells were infected as described above, but with MOI 10, and further incubated for 1 or 4 h. Infection was terminated and cells were fixed with 4% PFA overnight. The next day, cells were blocked in PBS containing 10% FCS and stained with anti-myeloperoxidase (MPO) antibody for 1 h, followed by 30 min incubation with the secondary antibody and subsequent mounting of the slides as described above. Images were collected with a Leica TCS SP5 confocal laser scanning microscope and processed using the Leica Application Suite (version 2.7.3.9723). Analysis of NET formation was performed by Fiji (58), calculating the ratio of MPO-positive (NET marker) and DAPI-positive (cellular DNA) signal in each condition.

### Cytokine Release Measurements

PMN were isolated and infected as described above. In these experiments, supernatants were kept and analyzed by Luminex^®^ (Austin, USA) for quantification of bactericidal/permeability increasing protein (BPI) and IL-8 as described earlier (60, 61). Mean fluorescence intensities (MFI) were acquired using the Luminex xMAP 100 system (Luminex Corp).

### Cell Viability Assays

Cell viability was assessed by Annexin V (BD Biosciences, #550475)/Propidium iodide (PI, Sigma-Aldrich, P4864) staining. PMN were isolated and infected as described above. Then, cells were washed and stained with Annexin V/PI, according to ‘BD Biosciences’ protocol. In detail, PMN were washed with cold PBS, adjusted to 10^6^ cells/ml in 1x Binding Buffer (10x, BD Biosciences, #556 454) and stained for 15 min. Analysis was performed using a FACS Canto II flow cytometer (BD). MFI were calculated by FlowJo software (version 10).

### Western Blotting

For analysis of pp38 abundance, PMN were lysed in RIPA buffer (25 mM sodium deoxycholate, 1% SDS, 0.4% EDTA, 10 mM NaF, 1% NP 40 in H_2_O_dd_) containing complete protease inhibitors (Roche, #1183617001) and PhosphoStop (Roche, #04906837001). After homogenization, proteins were separated on a 13% polyacrylamide gel and subsequently blotted on PVDF membranes (Merck, IPFL 00010). Images were acquired using Luminata Forte HRP substrate (Millipore, #WBLUF0500) on a Chemo Star imager (Intas).

### Mobility Assays

For the detection of spontaneous (undirected) movement, PMN were isolated as described above and seeded in 8-well IBIDI live cell chamber slides (200 000 cells per well). Chambers were incubated at 37°C, 5% CO_2_ for 1 h. Subsequently, 40 mM NaCl was added where indicated (= high salt, HS). After 1 h of additional incubation, cells were infected with *E. coli* at a multiplicity of 1 (MOI 1) and 10 ng/ml PMA was added where indicated. The chamber was immediately placed on a Leica SP5 confocal laser scanning microscope in a pre-heated (37°C) environment. Prior to and during the measurement, temperature was controlled by ‘The cube’ (Life Imaging Services), keeping the cell environment at 37°C. Cells were recorded for 30 min by acquiring pictures in 10 s intervals. Assessment of cell mobility was performed by Fiji plugin ‘manual cell tracker’ analyzing 19-20 cells per video which showed at least a minimal movement. PMN migration was not given in µm but in arbitrary units (a. u.) since our experimental setup does not allow for reliable detection of cell movement in the z-axis.

Direct PMN migration towards fMLP was analyzed with the QCM Chemotaxis Cell Migration Assay (Sigma, #ECM504) according to the manufacturers’ instructions. Briefly, PMN were isolated as described above and seeded in 24-well plates (500 000 cells per well) in serum-free medium. After 30 min incubation at 37°C, 5% CO_2_, 40 mM NaCl was added where indicated and cells were incubated further for 1 h. Subsequently, PMN were washed twice in RPMI medium containing 0.5% BSA and adjusted to 10^6^ cells/ml. Based on the Boyden chamber assay principle (62, 63), 250 µl cell suspension were added into each insert and 300 µl serum-free media ± 1 µM fMLP were added into the lower chamber. HS was added where indicated to each insert and lower chamber. The plate was incubated for 4 h at 37°C, 5% CO_2_, then the inserts were removed and 200 µl cell suspension with migrated cells was transferred from the lower chamber to a 96-well plate. 20 µl of WST-1 dye solution was added to each well and PMN were incubated for 2 h before fluorescence was measured using a microplate reader (Bio-Rad).

### Quantification of Reactive Oxygen Species

We used luminol-enhanced chemiluminescence for ROS quantification as described earlier (49). PMN were isolated as described above, seeded in 24-well plates (250 000 cells per well) and incubated for 30 min at 37°C, 5% CO_2_. Subsequently, where indicated, cells were treated with 3 µM DPI for additional 30 min. After that incubation period, 40 mM NaCl was added to the media and incubated for additional 1 h. Following this step, cells were transferred into FACS tubes, washed and resuspended in HBSS containing luminol (Sigma, #A8511) ± 40 mM NaCl and incubated for additional 15 min at 37°C, 5% CO_2_. ROS formation was induced upon stimulation with 1 µM fMLP and the measurement of ROS production using a Lumat^3^ LB 9507 Tube Luminometer (Berthold Technologies) was started (2 s interval). Where indicated, 10 ng/ml PMA was added simultaneously with fMLP.

### Flow Cytometry

After PMN isolation, 250 000 cells per well were seeded in 24-well plates and incubated at 37°C, 5% CO_2_ for 1 h. 40 mM NaCl was added to the HS condition and cells were incubated again for 1 h. Cells were subsequently infected with *E. coli* (MOI 1) for 1 h. Infection was stopped by addition of 500 µl cold PBS. Media including cells and bacteria were transferred into FACS tubes and centrifuged. Cells were resuspended in 500 µl FACS buffer (PBS containing FCS, EDTA and NaN_3_) and stained either with anti-CD35 (also known as complement receptor type 1, CR1) and anti-CD66b (carcinoembryonic antigen-related cell adhesion molecule 8, CEACAM8) or with anti-CD11b (complement receptor type 3, CR3) and anti-CD62L (L-Selectin) antibodies for 30 min at 4°C, respectively. Cells were washed and resuspended in 200 µl FACS buffer and recorded using a FACS Canto II flow cytometer (BD). MFI were calculated by FlowJo software (version 10).

### Statistics

All data are presented as means ± standard error of mean (s.e.m.) unless indicated otherwise. In all experiments (unless indicated otherwise), ‘n’ denotes separate wells from at least two independent experiments. Graphs and statistics were carried out by GraphPad Prism (v.6.0 or 8.0). In IL-8 quantifications and Boyden chamber assays, we used ROUT to identify outliers. Data was analyzed regarding normal distribution using the Kolmogorov-Smirnov test and compared by Student’s two-tailed t tests with Welch’s correction (for normally distributed data sets) or Mann-Whitney U tests (for non-normally distributed data sets), respectively. When two or more groups were compared, normally distributed data sets were analyzed by one-way ANOVA with Bonferroni’s multiple-comparison test, whereas the Kruskal-Wallis test with subsequent Dunn multiple-comparison test was used for non-normally distributed data. Area under the curve (AUC) calculations were carried out with the AUC tool of GraphPad Prism (v.6.0 or 8.0), as described earlier by Gagnon et al. (64). We considered p-values < 0.05 as statistically significant (*).

## Results

### High Extracellular Na^+^ Impairs Antibacterial Activity of Murine and Human Neutrophils

**Figure 1:**
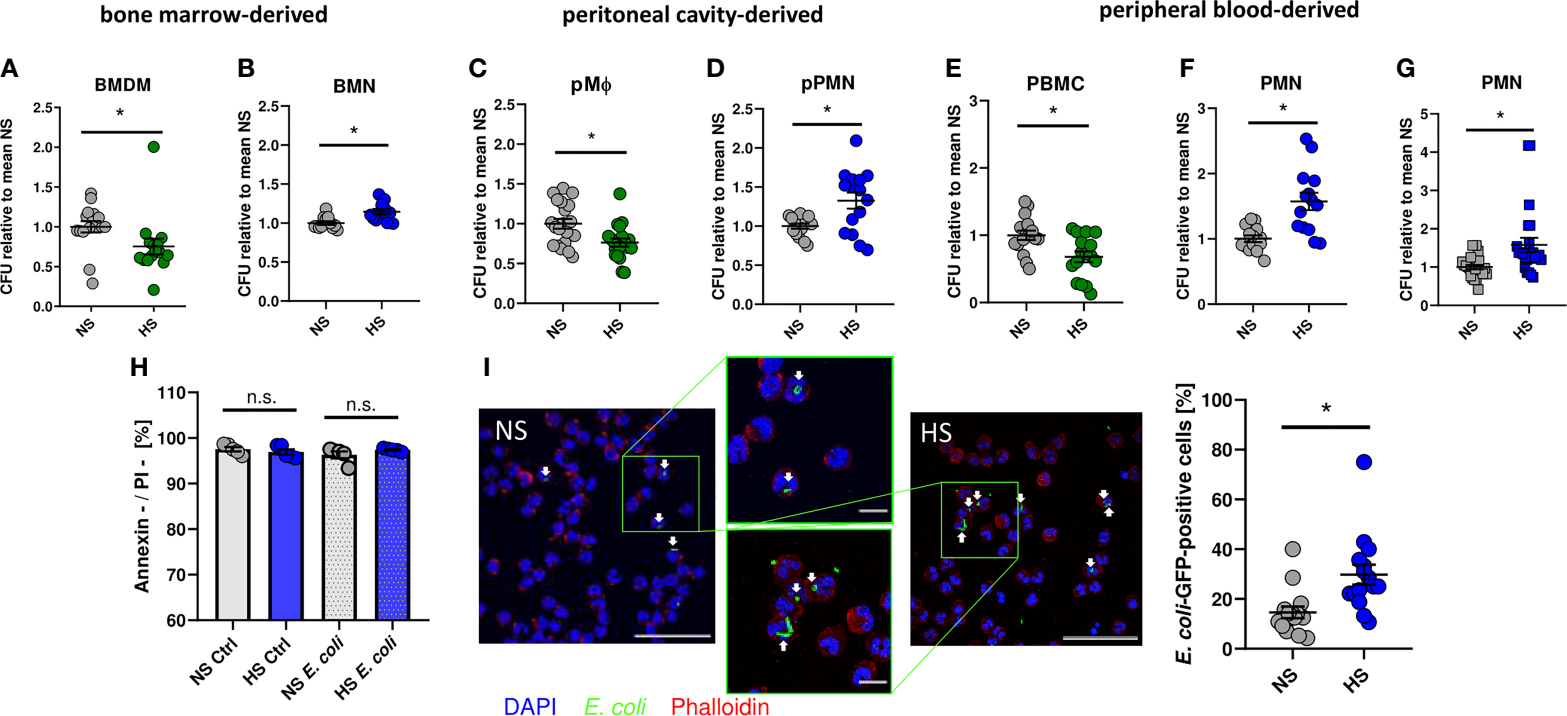
High salt impairs bacterial killing of neutrophils. Antibacterial activity of **(A)** murine bone marrow-derived macrophages (BMDM; means ± s.e.m.; n = 15-16; Mann-Whitney test; *p < 0.05), **(B)** murine bone marrow-derived neutrophils (BMN; means ± s.e.m.; n = 12; Mann-Whitney test; *p < 0.05), **(C)** murine peritoneal macrophages (pMΦ; means ± s.e.m.; n = 20; Student’s t test; *p < 0.05), **(D)** murine peritoneal neutrophils (pPMN; means ± s.e.m.; n = 15; Student’s t test with Welch’s correction; *p < 0.05), **(E)** human peripheral blood mononuclear cells (PBMC; means ± s.e.m.; n = 16; Student’s t test; *p < 0.05) and **(F)** human peripheral blood neutrophils (PMN, means ± s.e.m.; n = 14; Student’s t test with Welch’s correction; *p < 0.05) infected with *E. coli* under NS or HS (NS + 40 mM NaCl) conditions. **(G)** Antibacterial activity of PMN infected with *S. aureus* (means ± s.e.m.; n = 24; Mann-Whitney test; *p < 0.05). **(H)** Cell viability by Annexin/PI staining in uninfected (Ctrl) and *E.coli*-infected PMNs (means ± s.e.m.; n = 5; Student’s t test and Mann-Whitney test; n.s., not significant; *p < 0.05). **(I)** Infection rate in PMN 1 h after *E. coli*-infection; Bacterial load under NS or HS conditions, intracellular bacteria marked by arrowheads. A representative image out of three independent experiments is displayed. *E. coli-GFP*, green; Phalloidin, red; DAPI (DNA), blue. Scale bar: 50 µm (means ± s.e.m.; n = 15; Mann-Whitney test; *p < 0.05).

As demonstrated earlier (8, 12, 13, 65), addition of 40 mM NaCl to standard cell culture media (= high salt, HS) increased the antimicrobial activity of murine BMDM against *E. coli* (**Figure 1A**). In contrast, however, HS conditions impaired the antibacterial activity of bone marrow-derived murine neutrophils (BMN) (**Figure 1B**). Likewise, HS boosted antimicrobial activity of murine peritoneal macrophages (pMΦ; **Figure 1C**) and diminished the bactericidal activity of murine peritoneal elicited neutrophils (pPMN; **Figure 1D**). In accordance with these findings obtained from murine cells, HS augmented the antibacterial activity of human peripheral blood mononuclear cells (PBMC; **Figure 1E**) and, again, decreased the antibacterial activity of human PMN isolated from peripheral blood (**Figure 1F**). Moreover, HS not only impaired PMN clearance of *E. coli*, but also of Gram-positive *S. aureus* (**Figure 1G**). Of note, increases of osmolality and tonicity using mannitol did not impair the antibacterial activity of human PMN directed against *E. coli* or *S. aureus* (**Figure S1**). Annexin V/propidium iodide (PI) stainings of uninfected and infected PMN revealed no differences in the fraction of viable (Annexin V**^-^**/PI**^-^**) cells between NS or HS conditions (**Figure 1H**), suggesting that HS did not affect the cellular viability. In line with this, confocal microscopy revealed more GFP-labelled *E. coli* in infected PMN under HS conditions (**Figure 1I**). This indicates that, in contrast to macrophages, HS impairs the antibacterial activity of murine and human PMN.

### HS Exposure Impairs Neutrophil Mobility and Activation

**Figure 2:**
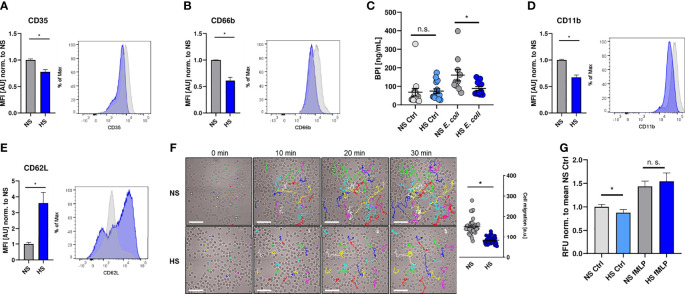
High salt diminishes cell activation and migration. **(A, B)** Flow cytometry analysis of surface expression of CD35 and CD66b on infected PMN 1 h post infection (means ± s.e.m.; n = 10; Student’s t test with Welch’s correction and Mann-Whitney test; *p < 0.05). **(C)** Extracellular bactericidal/permeability increasing protein (BPI) levels of control and infected (*E. coli*) PMN under NS or HS conditions (means ± s.e.m, n = 10-15; Mann-Whitney test; n.s., not significant; *p < 0.05). **(D, E)** As **(A, B)**, but surface expression of CD11b and CD62L was analyzed (means ± s.e.m.; n = 10; Student’s t test with Welch’s correction and Mann-Whitney test; *p < 0.05). **(F)** Spontaneous movement of PMN [given in arbitrary units (a. u.)] upon *E. coli*-infection under NS or HS conditions. Scale bar: 50 µm (means ± s.e.m.; n = 40; each dot represents individual cell traces from two independent experiments; Mann-Whitney test; *p < 0.05). **(G)** Spontaneous and directed movement towards fMLP of PMN measured by Boyden chamber assays (means ± s.e.m.; n = 15-17; Student’s t test and Mann-Whitney test; n.s., not significant; *p < 0.05).

In a next step, we investigated if the suppressive effect of HS is limited to their antimicrobial capacity or whether HS impairs additional PMN functions. For that purpose, we analyzed the expression of well-established cell surface markers to characterize neutrophil degranulation and activation status (66–70), which had been shown to be modified under HS conditions (40, 41, 43). HS interfered with the surface expression of CD35 (**Figure 2A**) and CD66b (**Figure 2B**), which are both markers for PMN degranulation (66, 67, 71, 72). This resulted in a reduced frequency of degranulated PMN under HS conditions (**Figure S2**). In line with this, HS conditions reduced the release of bactericidal/permeability increasing protein (BPI; **Figure 2C**), which is stored in primary (azurophilic) granules (73). HS conditions additionally diminished the expression of the complement receptor 3 (CR3, CD11b, **Figure 2D**), which is linked to PMN activation (68, 70, 74, 75). Moreover, HS-treated PMN displayed a higher surface expression of CD62L (**Figure 2E**; L-selectin), whose expression inversely correlates with the activation and migratory potential of PMN (76).

To analyze the impact of HS on the spontaneous movement capacity of PMN, we monitored the migratory trajectories of PMN using live cell imaging. Visualization and quantification of the migrated distance over 30 min revealed that HS conditions compromised the spontaneous movement of PMN (**Figure 2F**). Next, we performed Boyden chamber assays (62). In line with our previous findings, we found less spontaneous transmigration of HS-exposed PMN in the absence of a chemoattractant stimulus into the lower chamber. Interestingly, HS conditions did not impair the directed movement of PMN towards N-formylmethionyl-leucyl-phenylalanine (fMLP; **Figure 2G**) which is an established chemoattractant and proinflammatory activator of PMN (77–82). Taken together, these findings demonstrate that HS conditions not only interfere with the antimicrobial activity, but also diminish the overall activation status and spontaneous migratory capacity of PMN.

### Elevated Extracellular Na^+^ Levels Diminish ROS Production in Neutrophils

**Figure 3:**
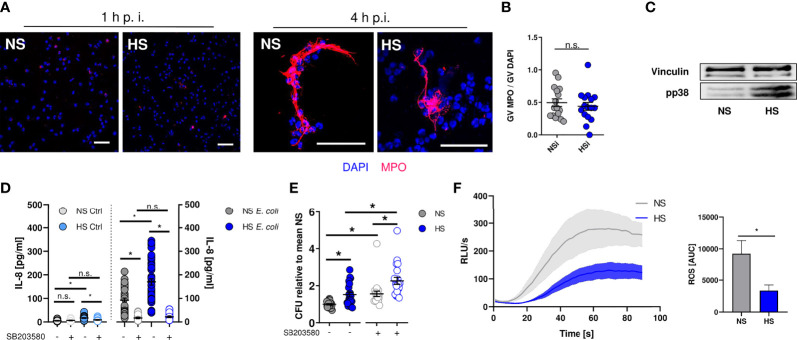
Elevated Na^+^ decreases ROS production, independent of NET formation or p38/MAPK signaling. **(A, B)** Staining of extracellular myeloperoxidase (MPO) **(A)** 1 h or **(B)** 4 h after *E. coli-*infection of PMN. A representative image out of three independent experiments is displayed. MPO, red; DAPI (DNA), blue. Scale bar: 50 µm. Gray value analysis of MPO positive NETs (means ± s.e.m.; n = 16; each dot represents an individual confocal image from three independent experiments; Student’s t test; n.s., not significant). **(C)** Representative phospho-p38/MAPK and vinculin immunoblot of PMN 30 min after *E. coli* infection out of three independent experiments. **(D)** IL-8 in the supernatant of control and infected PMN under NS or HS conditions ± p38-inhibitor SB203580 (means ± s.e.m.; n = 25-45; Kruskal-Wallis test with subsequent Dunn multiple-comparison test; n.s., not significant; *p < 0.05). **(E)** Antibacterial activity of PMN 1.5 h after *E. coli*-infection ± p38 inhibitor SB203580 (means ± s.e.m.; n = 21; Kruskal-Wallis test with subsequent Dunn multiple-comparison test; *p < 0.05). **(F)** ROS production upon fMLP stimulation; Luminometric ROS detection; time curve and AUC values (means ± s.e.m.; n = 5; Student’s t test; *p < 0.05).

Next, we set out to identify how HS alters the antimicrobial effector function of PMN. Neutrophils can trap and kill bacteria within neutrophil extracellular traps (NETs) [reviewed in: (83, 84)]. NETs are composed of neutrophil-derived cellular DNA, which serves as a scaffold for antimicrobial granule proteins such as the myeloperoxidase (MPO) (85). Extracellular MPO abundance can be used as a measure of NET formation (86). By confocal microscopy, we quantified extracellular MPO levels in infected PMN. In line with earlier findings (87), we did not detect NET formation at early time points (**Figure 3A**) under NS and HS conditions, while there was robust NET formation at 4 h after infection which was independent of extracellular Na^+^ levels (**Figure 3B**). Hence, we conclude that HS conditions did not affect NET-dependent antimicrobial activity of PMN.

In macrophages and PMN, exposure to HS augments the activation of the osmoprotective p38/MAPK signaling pathway (8, 35). In accordance to these reports, we found that HS enhanced the phosphorylation of p38/MAPK in neutrophils (**Figure 3C**). HS-boosted p38/MAPK was linked to IL-8 release in PBMC (88). In line with this, we found that HS increased the IL-8 release from PMN (**Figure 3D**). Since p38/MAPK was required for HS-boosted antimicrobial function in macrophages (8, 12), we wanted to assess bacterial elimination in PMN in the absence or presence of the pharmacological p38-inhibitor SB203580. Inhibition of p38/MAPK largely abolished HS-boosted IL-8 release (**Figure 3D**) and, in line with earlier findings (89, 90), impaired the antimicrobial activity of PMN under NS and HS conditions (**Figure 3E**). However, inhibition of p38/MAPK did not abolish the blunted antimicrobial activity under HS conditions (**Figure 3E**). This suggests that p38/MAPK-dependent signaling does not play a role in HS-impaired antimicrobial activity of PMN.

PHOX-dependent ROS production critically contributes to the antimicrobial activity of neutrophils [reviewed in: (33, 91)]. We measured ROS production for 90 s after fMLP-stimulation using continuous luminol measurements that allow for immediate detection of ROS over time (49). These assays revealed that HS conditions substantially diminished fMLP-triggered ROS production (**Figure 3F**), demonstrating that HS-mediated impairment of antibacterial PMN activity is independent of osmoprotective signaling and NET formation, possibly due to reduced ROS production.

### Disturbed ROS Production Underlies Impaired Antimicrobial Activity in PMN Under HS Conditions

**Figure 4:**
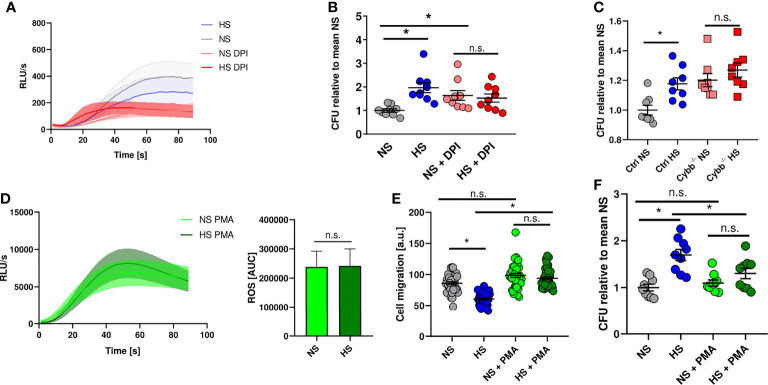
NADPH oxidase inhibition mimics high salt effect while PMA abrogates high salt-induced killing deficiency. **(A)** ROS production in DPI pre-treated PMN under NS or HS conditions, stimulated with fMLP (means ± s.e.m.; n = 4-5). **(B)** Antibacterial activity of PMN 1.5 h after *E. coli*-infection ± PHOX-inhibitor (DPI; means ± s.e.m.; n = 9; Kruskal-Wallis test with subsequent Dunn multiple-comparison test; n.s., not significant; *p < 0.05). **(C)** Antibacterial activity of neutrophils from PHOX-deficient (*Cybb* ^-/-^) and control mice 1.5 h after *E. coli*-infection (means ± s.e.m.; n = 8; Mann-Whitney test; n.s., not significant; *p < 0.05). **(D)** ROS production in fMLP/PMA-stimulated PMN (PMA) under NS and HS conditions; time curve and AUC values (means ± s.e.m.; n = 8; Student’s t test; n.s., not significant). **(E)** Cell migration (given as arbitrary units) upon PMA stimulation of *E. coli*-infected PMN under NS and HS conditions (means ± s.e.m.; n = 39-40; Kruskal-Wallis test with subsequent Dunn multiple-comparison test; n.s., not significant; *p < 0.05). **(F)** Antibacterial activity of PMN 1.5 h after *E. coli*-infection ± PMA-stimulation under NS or HS conditions (means ± s.e.m.; n = 9; ordinary one-way ANOVA with Bonferroni’s *post hoc* test; n.s., not significant; *p < 0.05).

Therefore, we analyzed whether blocking ROS production in PMN using diphenyleneiodonium chloride (DPI) (92) mimics HS-induced attenuation of antibacterial activity. DPI-treatment diminished ROS production in PMN stimulated with fMLP under NS conditions (**Figure 4A**) and reduced the antibacterial activity of PMN under NS to levels obtained for PMN exposed to HS conditions (**Figure 4B**). Of note, HS did not further compromise the ability of PMN to fight *E. coli* (**Figure 4B**). This suggests that HS-triggered impairment of PHOX activity underlies the blunted antimicrobial PMN response. To corroborate this finding, we purified neutrophils from bone marrow of mice lacking the cytochrome b-245 subunit of PHOX (*Cybb^-/-^*). We infected these cells with *E. coli* and analyzed their bactericidal activity. These experiments confirmed the previous findings using the pharmacological inhibitor DPI (**Figure 4C**). In a second approach, we wanted to test if increasing ROS production using phorbol-12-myristate-13-acetate (PMA) is able to rescue HS-impaired antimicrobial capacity of PMN. PMA is known to boost ROS production in PMN (92, 93). After additional stimulation with PMA, there was a robust increase in ROS production which was unaffected by HS exposure (**Figure 4D**). PMA-treatment normalized the blockade of HS on the migratory potential of PMN (**Figure 4E**). Of utmost importance, addition of PMA abolished the inhibitory effect of HS on the antimicrobial activity of PMN (**Figure 4F**). We conclude that HS-triggered impairment of ROS production in PMN underlies their disturbed antimicrobial activity under HS conditions.

## Discussion

Here, we demonstrate that a Na^+^-rich environment curtails the expression of activation markers, spontaneous movement, and ROS production of PMN upon exposure to bacteria and bacterial products which ultimately leads to diminished antimicrobial capacities of PMN. In contrast, simultaneous infection and increase of extracellular Na^+^ reportedly triggers more ROS production in PMN and enhances antibacterial activity of PMN in this experimental system (36). Moreover, Junger et al. reported that exposure of PMN to HS after fMLP-stimulation did not affect ROS production (35). However, in line with our findings, other groups showed that stimulation of PMN after exposure to HS conditions caused disturbed assembly of PHOX (38), reduced ROS production (35, 37, 39), decreased expression of various surface markers related to human neutrophil activation (39–41, 43), and bacterial killing (46, 47). These findings suggest that the sequence of stimulation and Na^+^ exposure critically influences the outcome of PMN activation.

If PMN are exposed to very high Na^+^ environments (addition of 130 mM NaCl) this reportedly impaired migration towards an established chemoattractant (44). We, however, increased the Na^+^ concentrations by addition of 40 mM NaCl in order to simulate Na^+^ conditions that can be encountered in inflamed and infected skin tissues (7, 8, 10, 11). Although we found a reduced spontaneous migration of PMN upon exposure to increases in Na^+^, these HS conditions did not affect chemotaxis. This indicates that local Na^+^-rich environments that can be present in infected skin still allow for PMN infiltration into tissues if a chemoattractant such as fMLP is present. Since inflamed and infected skin tissues can display increased Na^+^ levels (7, 8, 10, 11), it is likely that PMN pass through Na^+^-rich environments before reaching the invading pathogen. Therefore, our findings might have relevance in migration of PMN through Na^+^-rich environments and PMN-dependent clearance of bacteria from tissues.

Here, we confirm findings that HS impairs the antibacterial activity of PMN (46, 47). These studies (46, 47), however, assessed the effects of very high Na^+^ levels that are present in the renal medulla (94). We, however, simulated moderate increases in Na^+^ that can be encountered in inflamed and infected skin tissues (7, 8, 10, 11). Moreover, our studies provide mechanistic insights into how HS impairs antibacterial responses in PMN by pinning down antibacterial effectors disturbed by the addition of Na^+^. In contrast to an earlier report (95), we did not detect any difference in viability upon HS exposure in our experimental setup. Therefore, we exclude cytotoxicity as cause of reduced antimicrobial activity. Further we interrogated processes and pathways relevant for microbial disposal in PMN. Oxidative killing is promptly triggered in PMN following microbial internalization. We observed reduced ROS release in HS-exposed PMN. Using pharmacological PHOX inhibitors, neutrophils from *Cybb* ^-/-^ mice, and pharmacological means to rescue HS-blunted ROS production, we demonstrated that HS impairs the antibacterial activity by dampening PHOX-dependent ROS production. From this finding, we would predict that the role of HS on the outcome of PMN-pathogen interaction depends on the susceptibility of the pathogen to ROS-dependent killing mechanisms. Indeed, HS did not affect the *in vitro* antimicrobial activity of PMN isolated from bone marrow directed against an uropathogenic *E. coli* (UPEC) (25), which is known to suppress oxidative burst in PMN and to be more resistant to reactive oxygen species than commensal strains (96). Moreover, in line with this reasoning, we found that HS diminished the antimicrobial control of PMN directed against *S. aureus*, whose control depends on PHOX-dependent ROS production (97).

NADPH oxidase-dependent ROS production [reviewed in: (98)] may trigger NET production or alternatively NETs require p38/MAPK (89, 99). Nadesalingam et al, who also demonstrated that increasing Na^+^ availability decreases ROS production in PMN, showed that this is linked to impairment of NET formation (45). In our study, however, HS did not affect generation of NETs in PMN 4 h after *E. coli-*infection, indicating that ROS levels are not critical for casting of NETs in our experimental setup. Thus, a direct link of NET formation to HS-impaired antibacterial activity in our experiments is unlikely.

p38/MAPK activation contributes to cytokine release (including IL-8) by neutrophils (100), NADPH oxidase-dependent ROS production (101, 102) and subsequent antimicrobial activity of neutrophils under NS conditions (89, 90). In line with this, we found that p38/MAPK inhibition reduced antimicrobial activity of PMN and diminished HS-triggered or boosted IL-8 release. However, HS conditions diminished the antimicrobial activity of PMN in the absence or presence of pharmacological p38/MAPK blockade. This strongly suggests that the HS-impaired antimicrobial activity is not linked to HS-triggered modulation of p38/MAPK activity in PMN.

In sum, HS-mediated impairment of antimicrobial activity is due to blunted PHOX-dependent ROS production. The molecular mechanism that results in impaired PHOX-dependent ROS production requires further investigation. Our data strongly suggest that impaired ROS production is uncoupled from HS-modified p38/MAPK. It is possible that increases in Na^+^ directly impair enzymatic PHOX activity. Since increases in Na^+^ are known to affect the dynamics of the actin cytoskeleton (41) and Rac proteins that interact with the actin cytoskeleton [reviewed in: (103)] are required for proper PHOX activity [reviewed in: (104)], it is also possible that molecules involved in PHOX assembly, e.g. Rho GTPases, or rearrangement of the actin cytoskeleton could underlie our finding.

We published earlier that HS conditions led to impaired ROS production upon *E. coli* infection in macrophages as well, which nevertheless showed increased antibacterial activity under HS conditions (12). In contrast to PMN, PHOX-dependent ROS production was dispensable in HS-augmented antibacterial activity in macrophages (12). Instead, HS boosted autophagy and autolysosomal targeting of bacteria for degradation into these highly acidic compartments (12, 13). This demonstrates that HS differentially alters antibacterial responses in distinct phagocytes.

Macrophages play an important role in controlling tissue integrity and resolution of inflammation by eliminating intruding invaders and fostering the degradation of debris (105–107). Neutrophils are the first responders that are attracted to tissue injuries, which cannot be managed by local tissue-resident macrophages already on site (108). While neutrophil accumulation helps in warding off infections, this comes at a cost of collateral tissue damage. Although it is established that in inflamed and infected tissue Na^+^-rich microdomains can be present (7, 8, 10), it is unclear which mechanisms orchestrate Na^+^ accumulation [reviewed in: (14)] and when Na^+^ accumulation in tissues appears in the course of inflammation and infection. It is very tempting to speculate that Na^+^-rich environments are a signature of prolonged inflammatory processes. Likewise it is possible that within inflamed/infected tissues Na^+^ gradients exist that modulate immune cell activity. Therefore, interstitial Na^+^-accumulation could represent an 'ionic checkpoint' (20) which shapes a niche that ensures tissue integrity by dampening PMN function on the one hand, while boosting macrophage-dependent antibacterial activity and clearance of tissue debris on the other hand.

In line with this hypothesis, several reports showed that hypertonic saline infusion is linked to reduced lung injury and concomitant decreased neutrophil activation (37, 109, 110) as well as increased bacterial removal by immune cells in the peritoneal cavity (37). Further studies are required to systematically assess the role of local tissue Na^+^ in the resolution of inflammatory diseases. Our findings provide new views on the resolution process, which have direct clinical relevance, and open new lines of investigations.

## Data Availability Statement

The raw data supporting the conclusions of this article will be made available by the authors, without undue reservation.

## Ethics Statement

The studies involving human participants were reviewed and approved by Ethikkommission bei der Universität Regensburg. The patients/participants provided their written informed consent to participate in this study. The animal study was reviewed and approved by Animal Welfare Committee of the local governmental authorities (Regierung von Unterfranken Würzburg, Germany).

## Author Contributions

LK, KB, SE, PN, TO, AW, VS, MT, and AS acquired, analyzed, and interpreted data. MH, MS, and AD interpreted data and contributed to the design of experiments. JJ interpreted data, designed, conceptualized and oversaw the study. LK provided the first draft of the manuscript. Manuscript was revised by TO, AS, VS, SE, KB, PN, MH, MS, AD, and JJ. All authors contributed to the article and approved the submitted version.

## Funding

JJ was supported by Deutsche Forschungsgemeinschaft [German Research Foundation, DFG, JA 1993/4‐1, SFB1350 (project nr. 387509280, TPB5)] and by the Bavarian Ministry of Science and the Arts in the framework of the Bavarian Research Network ‘New Strategies Against Multi-Resistant Pathogens by Means of Digital Networking–bayresq.net’. MH received funding from the DFG (DFG-FOR2886 PANDORA Project B3).

## Conflict of Interest

The authors declare that the research was conducted in the absence of any commercial or financial relationships that could be construed as a potential conflict of interest.

## Publisher’s Note

All claims expressed in this article are solely those of the authors and do not necessarily represent those of their affiliated organizations, or those of the publisher, the editors and the reviewers. Any product that may be evaluated in this article, or claim that may be made by its manufacturer, is not guaranteed or endorsed by the publisher.
