# Supplementary figures and images for: High Na+ Environments Impair Phagocyte Oxidase-Dependent Antibacterial Activity of Neutrophils
Source: Front Immunol. 2021 Sep 10;12:712948. doi: 10.3389/fimmu.2021.712948 (PMC8461097; doi:10.3389/fimmu.2021.712948)

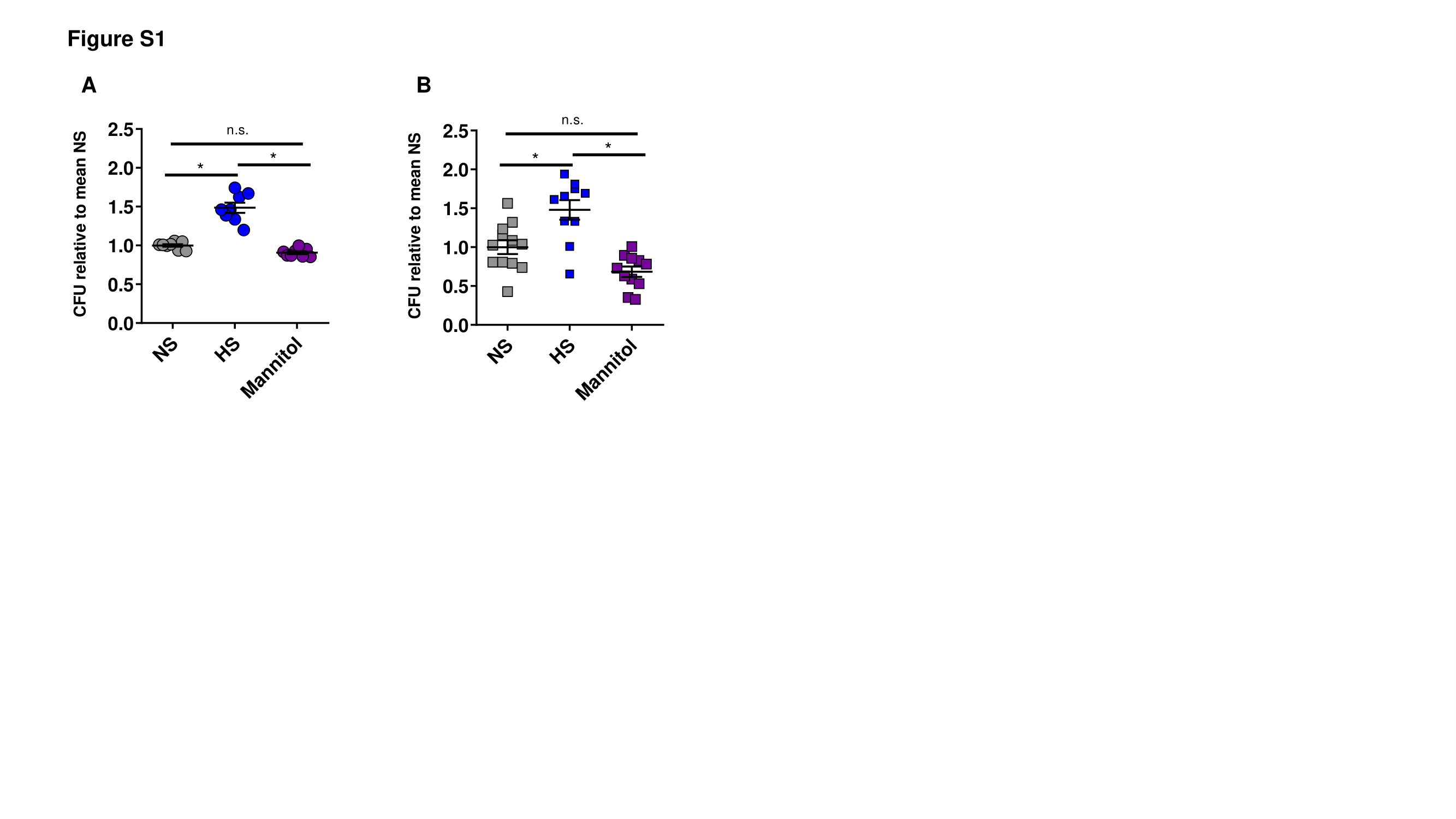

Supplement: Supplementary file 1 [file Image_1.tiff]

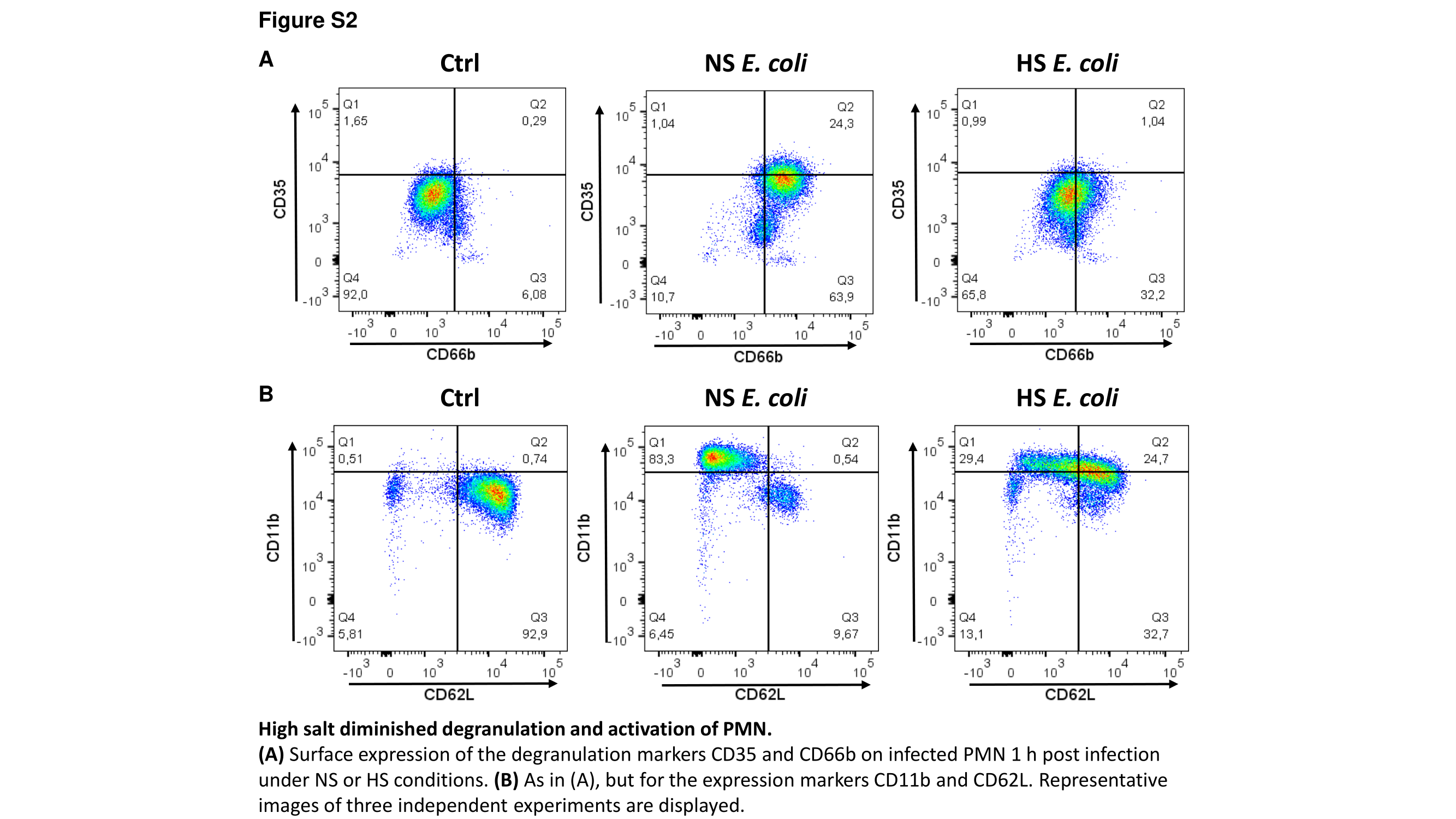

Supplement: Supplementary file 2 [file Image_2.tiff]
